# Estimating the prevalence of dementia using multiple linked administrative health records and capture–recapture methodology

**DOI:** 10.1186/s12982-017-0057-3

**Published:** 2017-02-27

**Authors:** Michael Waller, Gita D. Mishra, Annette J. Dobson

**Affiliations:** School of Public Health, University of Queensland, Brisbane, QLD 4006 Australia

**Keywords:** Dementia, Prevalence, Incidence, Linked data, Capture–recapture

## Abstract

**Background:**

Obtaining population-level estimates of the incidence and prevalence of dementia is challenging due to under-diagnosis and under-reporting. We investigated the feasibility of using multiple linked datasets and capture–recapture techniques to estimate rates of dementia among women in Australia.

**Methods:**

This work is based on the Australian Longitudinal Study on Women’s Health. A random sample of 12,432 women born in 1921–1926 was recruited in 1996. Over 16 years of follow-up records of dementia were obtained from five sources: three-yearly self-reported surveys; clinical assessments for aged care assistance; death certificates; pharmaceutical prescriptions filled; and, in three Australian States only, hospital in-patient records.

**Results:**

A total of 2534 women had a record of dementia in at least one of the data sources. The aged care assessments included dementia records for 79.3% of these women, while pharmaceutical data included 34.6%, death certificates 31.0% and survey data 18.5%. In the States where hospital data were available this source included dementia records for 55.8% of the women. Using capture–recapture methods we estimated an additional 728 women with dementia had not been identified, increasing the 16 year prevalence for the cohort from 20.4 to 26.0% (95% confidence interval [CI] 25.2, 26.8%).

**Conclusions:**

This study demonstrates that using routinely collected health data with record linkage and capture–recapture can produce plausible estimates for dementia prevalence and incidence at a population level.

**Electronic supplementary material:**

The online version of this article (doi:10.1186/s12982-017-0057-3) contains supplementary material, which is available to authorized users.

## Background

In Australia, it is estimated that 9% of people aged over 65, and 30% of those aged over 85 have dementia [1]. However, many of the estimates of dementia prevalence have been based on older datasets drawn from other countries [2], or from a single small area data set [1].

Despite expected increases in the number of people with dementia due to population ageing, there is some evidence that the age-specific incidence rates of dementia in first world countries may be declining as more recent generations are reaching old age [3–5], possibly because of increased education [6–8], more stimulating environments [9], and advances in the control of vascular risk factors [4, 10]. Due to these competing age and cohort effects, a more complete understanding of how the case load of dementia is changing over time is required, for example, for public policy. Methods to obtain accurate and current estimates of rates of dementia are necessary to assess the health service needs of the elderly at a population level.

The Australian Longitudinal Study on Women’s Health (ALSWH) is a prospective national survey [11]. Three-yearly surveys and linked administrative records present an opportunity to estimate the overall incidence and prevalence of dementia using capture–recapture methods [12]. This approach has rarely been used before for dementia and not on a national level [13, 14].

An assessment of the value of these methods is important, as there are no standard population based surveillance systems for dementia using routinely collected data, and under-diagnosis and under-reporting of people living with dementia are well established [15].

The aim of this study is to demonstrate the use of this approach to obtain an accurate and up to date estimate of dementia rates in Australian women.

## Methods

Data from 12,432 women born between 1921 and 1926 (estimated response rate 37–40%), who responded to the ALSWH baseline survey in 1996, were used as a starting point to assess rates of dementia [16, 17].

The ALSWH is a nationally representative study which includes women from every Australian State and Territory [11]. The study sample was selected by Medicare Australia, the universal health care insurance scheme. Sampling was random, with women from rural and remote areas sampled at twice the rate of women in urban areas to facilitate comparisons between these groups [17]. The ALSWH sample of older women was generally representative of Australian women of the same age, but did include more women who were married or living with their partner, and more women with post-school qualifications, compared to the 1996 Australian Census [11, 16]. Each participant has a unique Medicare identification number which is used in some, but not all, administrative data sources thereby enabling deterministic record linkage.

### Data sources

Five data sources were used to identify records of dementia and Alzheimer’s disease in these women between 31 May 1996 and 6 March 2012 (the dates of first and last full surveys received from this cohort). We will refer to these as ‘dementia’ records throughout the paper.

### Self-reported survey data (A)

The survey data consisted of six surveys which occurred at 3-year intervals. Participants (or their proxies) were asked in Surveys 2–6 whether they had been diagnosed with or treated for dementia. Surveys 4 and 5 contained a free-text field where participants (or proxies) could explain reasons why they needed help to complete the survey. This text was searched for the terms ‘Alzheimer’s and ‘dementia’. Information on self-reported medication collected from Survey 4, and coded using the Anatomical Therapeutic Chemical index [18], was also used to identify women who used anti-dementia drugs (Additional file 1: Table A1). The date of survey response was used as a date of notification for each identified case. Notifications of dementia from this source commenced in 1999 (Survey 2 onwards).

### Aged care assessments (B)

Aged care assessment data were obtained from the Australian Institute of Health and Welfare [1], who extracted records for all ALSWH participants in the 1921–1926 cohort. As not all aged care records included the Medicare number, probabilistic linkage methods were used [19]. The matching process employed both name based linkage and key based linkage techniques. These linkages to the ALSWH data were estimated to have a sensitivity over 94% and a positive predictive value above 96% (AIHW communication). There were several sources used to identify dementia records: the Extended Care at Home Dementia Program; the Aged Care Assessment Program (which assesses the care needs of older people and assists in the access of appropriate types of care); and the Aged Care Funding Instrument (which assesses care needs as a basis for calculating and allocating funds to the aged care facility). As part of the Aged Care Assessment Program and the Aged Care Funding Instrument, diagnostic codes of dementia were recorded (Additional file 1: Table A1). These diagnoses were obtained through referrals to a general practitioner, geriatrician or psycho-geriatrician, or through an assessor (with consent) accessing medical history information from a relevant doctor. Each record had a date of service or assessment. Notifications of dementia from this source were available from July 2003.

### Causes of death (C)

Information on date and multiple causes of death was obtained from the National Death Index and the National Mortality Database [20]. Probabilistic matching, using names, date of birth and gender, was used to identify deaths among ALSWH participants [21]. Records of dementia or Alzheimer’s disease were identified using ICD9 and ICD10 codes (Additional file 1: Table A1).

### Pharmaceutical Benefits Scheme (D)

Information on drug prescriptions filled was obtained from Pharmaceutical Benefits Scheme records which cover all medications dispensed and/or subsidised under the universal national health insurance scheme [22]. Deterministic linkage of records for all ALSWH participants was conducted using their unique Medicare numbers [23]. This data source included prescription details, but not the reason for the prescription, for all subsidised prescriptions from July 2002 to June 2012. For women in this age group most prescriptions are subsidised, so the medication records are likely to be complete. The medications were coded using the ATC index [18] (Additional file 1: Table A1).

### Admitted patients hospital data (E)

Hospital admissions data were available from three Australian States (New South Wales, Queensland and South Australia). These data were extracted by health data linkage units in these jurisdictions using probabilistic matching [24–26]. Date of admission and doctor assigned diagnoses, coded using ICD10, were recorded [27]. The codes which indicated dementia or Alzheimer’s disease are provided in Additional file 1: Table A1. This data source included admissions from June 2000.

### Statistical analysis

The linked data were used to identify the total number of women with dementia records (from any of the available data sources), and to assess the overlap between these sources. The hospital data were not included in the primary analysis because these data were only available for three Australian States. Poisson regression was used to estimate the number of women with dementia who were not identified from any of the four (or five) sources [12]. The outcome of the model was the count of women with dementia identified from each combination of sources. The independent variables were indicators (1/0) for each data source, and possible interactions between these sources. The estimated number of ‘unidentified’ women with dementia was the exponent of the constant term in the Poisson model.

With four sources (i.e., self-reported survey, aged care assessments, causes of death and pharmaceuticals) there were 113 possible log-linear models [12]. Model averaging was used to obtain a weighted estimate of the number of unidentified women with dementia [28, 29]. This technique weights estimates from each model based on how well it fits the data, and then uses these weights to create an average estimate [Additional file 1: Table A2 (equations A1–A5)].

An overall estimate of the number of ‘unidentified’ women with dementia was calculated. Separate estimates for each age group were also produced and a pooled total was obtained (Additional file 1: Table A2, equations A6–A8). The following age groups (based on numbers of records) were used: 68–78, 79–80, 81–82, 83–84, 85–86 and 87–91, which ensured that almost all combinations of data sources were used for each model. If no records were identified from a specific combination of sources, a correction factor of (0.5)^g−1^ was added to that cell (where g is the number of sources) [12]. If records were identified from different sources in different age groups, the earliest date of a dementia record was used.

One of the assumptions of the capture–recapture method is that the population is closed, meaning that no individuals can migrate into or out of the study or be lost because of death [12]. In this analysis no new women entered the cohort, however, 5453 women died over the duration of follow-up and emigrations were possible, though unlikely. An adjustment was made to each estimate of the number of ‘unidentified’ women with dementia to account for those who died. This adjustment was based on the median date of death in each age group (Additional file 1: Table A2, equation A6). A 95% confidence interval [CI] for the estimated number of women with dementia from the capture–recapture analysis was produced. This confidence interval adjusts for sampling variation, and does not represent uncertainty regarding model assumptions [12].

The effect of including the hospital data as a fifth source was assessed in an analysis limited to the three States for which hospital data were available. In this analysis, four source and five source capture–recapture models were fitted and the results compared. Using five sources 6893 possible log-linear models were considered.

Prevalence and incidence rates were calculated by single year of age and then collapsed into 5-year age groups. For women identified with dementia from any of the sources, the earliest date of notification, date of birth and date of death were used in the calculation of prevalence and incidence rates. Deaths that occurred in any year might reduce the number women living with dementia in the numerator of the rate and would reduce the total number at risk in the denominator in both the prevalence and incidence calculations. For the ‘unidentified’ women living with dementia we knew the age group in which the diagnosis was estimated to have occurred, however, we did not have a date of death. To include these ‘unidentified’ women living with dementia in the prevalence and incidence calculations, for each age group (68–78, 79–80, etc.) a diagnosis of dementia was randomly assigned to the same number of women who were still alive at that age and did not have a record of dementia from any source. Additional records based on the percentage increase in age specific estimates, due to the inclusion of the hospital data in the five-source analysis, were also assigned in this way. This process was repeated 10 times to examine how the random allocation of the ‘unidentified’ women with dementia changed the results.

In all the analyses we assumed that all records of dementia reflect a participant’s true dementia status, and that a proportion of those without a record of dementia may also have dementia (i.e., the ‘unidentified’ cases).

## Results

A total of 2534 out of 12,432 (20.4%) women were identified as having dementia in at least one of the four main data sources (Table 1). The largest number of dementia records was identified from the aged-care assessments (2010 women, 16.2% of all the women and 79.3% of those with dementia records). Of the women with a record of dementia from the aged-care assessments, 65% had the dementia recorded more than once within this source. The source yielding the smallest number of dementia records was the self-reported survey data (18.5% of records). Of these self-reported records, 17.3% were reported with the help of a proxy, while the death certificates and pharmaceutical data had 31.0 and 34.6% of records respectively. In the States where hospital data were available, 55.8% of women with dementia were identified in this data source. There were 50 women (0.4% of all the women) with records in all four of the nationally available datasets, and 1329 (10.6% of all women) from one source only (Table 2).

**Table 1:** Demographic characteristics by source of data on dementia

	Self-reported survey	Aged care data	Causes of death	Pharmaceutical/prescription	Hospital data
Total identified cases	A	B	C	D	E
N = 2534	N = 468	N = 2010	N = 786	N = 877	N = 983
% of identified cases^b^	18.5%	79.3%	31.0%	34.6%	55.8%
Median age in years (quartiles)^a^	83.2 (79.9, 86.2)	85.1 (83.1, 86.8)	85.0 (82.8, 86.8)	82.7 (80.4, 85.2)	84.3 (81.8, 86.6)

Age (years)^a^	n (%)	n (%)	n (%)	n (%)	n (%)
<75	18 (3.8)	0	7 (0.9)	0	1 (0.1)
75–79	108 (23.1)	85 (4.2)	55 (7.0)	172 (19.6)	155 (15.8)
80–84	174 (37.2)	889 (44.2)	326 (41.5)	440 (50.2)	404 (41.1)
85–89	164 (35.0)	1009 (50.2)	379 (48.2)	259 (29.5)	403 (41.0)
≥90	4 (0.9)	26 (1.3)	11 (1.4)	6 (0.7)	20 (2.0)
Unknown		1 (0.05)	8 (1.0)		

A = Self-reported survey data

B = Aged care data

C = Death certificate data

D = Prescription data

E = Hospital admission patients data (only for Queensland, New South Wales, and South Australia; Total n = 7750)

^a^Age dementia was first recorded

^b^Row percentages do not add up to 100% because cases can be identified from more than one source

**Table 2:** Number of new records of dementia by age, as identified by different combinations of four data sources (n = 12,432)

	Age						
	68–78	79–80	81–82	83–84	85–86	87–91	Total
A	27	17	12	10	13	20	99
B	9	37	81	204	283	289	903
C	29	35	45	41	37	24	211
D	8	11	22	27	24	24	116
AB	11	14	17	25	37	17	121
AC	22	11	2	3	2	1	41
AD	4	2	1	2	4	5	18
BC	6	33	48	77	57	33	254
BD	24	54	76	98	84	51	387
CD	12	10	6	3	1	0	32
ABC	7	5	5	6	2	3	28
ABD	10	29	20	20	16	9	104
ACD	2	2	2	0	1	0	7
BCD	19	44	54	28	18	0	163
ABCD	16	16	12	3	3	0	50
Identified number	206	320	403	547	582	476	2534
Estimated extra	30	41	116	128	164	217	728
Total = identified + extra	236	361	519	675	746	693	3262
95% CI	201, 272	342, 380	411, 627	553, 797	634, 857	537, 848	2823, 4370
Adjustment for deaths	1 − (26/206) = 0.87	1 − (25/320) = 0.92	1 − (34/403) = 0.92	1 − (45/547) = 0.92	1 − (37/582) = 0.94	1 − (47/476) = 0.90	
							Pooled estimate 95% CI
							3229 2976, 3482

Correction factor of 0.125 added to cells which contain no identified cases

A = Self-reported survey data

B = Aged care data

C = Death certificate data

D = Prescription data

Using capture–recapture methods we estimated that there were 695 ‘unidentified’ women with dementia. Therefore the estimated total number of women with dementia was 3229, 95% CI (2976, 3482) or 26.0%, 95% CI (25.2, 26.8%) (cumulative incidence above the age of 70) (Table 2). The difference between the number of identified women with dementia (2534) and the capture–recapture estimate (3229) suggests that only using the available datasets would have underestimated the number of women with dementia by 27% (695/2534). The correction used on cells with no records had only a marginal effect on the estimates presented, as did the adjustment for deaths in each age category (Table 2). The effect of including the hospital data was assessed by restricting the analyses to women in New South Wales, Queensland and South Australia. The inclusion of the hospital data increased the estimated total percentage of women who had dementia slightly to 26.9%, 95% CI (26.0, 27.9%) (Additional file 1: Tables A3 and A4).

The average length of time to death or the end of follow-up was 13.0 years [standard deviation (SD) 4.1], and the average time to dementia, death or end of follow-up period was 11.3 years (SD 3.2). The prevalence and incidence of dementia are underestimated for the ages 70–79 because only self-reported survey data and cause of death data were available for the period 1996–2000. Using the capture–recapture estimates, rates of prevalence and incidence of dementia for ages 85+ are approximately double than in the ages 80–84 estimates (Table 3). The prevalence and incidence estimates changed only slightly when the ten different random allocations of ‘unidentified’ dementia records in each age group were used (data available on request).

**Table 3:** Prevalence and incidence of dementia by age

Age	Prevalence: identified dementia cases				Prevalence: estimated dementia cases^a^			
	Identified dementia cases	N	Prevalence %	95% CI	Estimated dementia cases^a^	N	Prevalence %	95% CI
70–74^b^	25	11,846	0.2	(0.1, 0.3)	37	11,846	0.3	(0.2, 0.4)
75–79^b^	352	12,029	2.9	(2.6, 3.2)	450	12,029	3.7	(3.4, 4.1)
80–84	1385	10,803	12.8	(12.1, 13.5)	1794	10,803	16.6	(15.9, 17.3)
85+	1946	8817	22.1	(21.2, 22.9)	2735	8817	31.0	(30.0, 32.0)

	Incidence: identified dementia cases				Incidence: estimated dementia cases^a^			
	Identified dementia cases	Person-years	Incidence rate per 100 person-years	95% CI	Estimated dementia cases^a^	Person-years	Incidence rate per 100 person-years	95% CI
70–74^b^	25	29,225.81	0.09	(0.06, 0.13)	37	29,210.15	0.13	(0.09, 0.17)
75–79^b^	335	56,712.76	0.59	(0.53, 0.66)	423	56,560.95	0.75	(0.68, 0.82)
80–84	1116	46,569.31	2.40	(2.33, 2.54)	1456	45,676.17	3.19	(3.02, 3.34)
85+	1058	22,076.41	4.78	(4.51, 5.09)	1532	20,596.46	7.44	(7.07, 7.82)

^a^Estimated cases = identified cases + estimated extra cases (based on inclusion of hospital data)

^b^Prevalence and incidence for ages 70–74 and 75–79 are underestimated because only self-reported data and cause of death data were available for the period 1996–2000

Dementia prevalence and incidence rates from the ALSWH study, compared to estimates from other international studies for women aged 80–85 and 85–89, are presented in Table 4. The prevalence and incidence rates of dementia for women aged 80–84 and 85+, based on identified records were broadly consistent with those reported previously. In contrast, estimates based on the capture–recapture techniques were higher than previously published prevalence and incidence figures (Table 4).

**Table 4:** Female prevalence and incidence of dementia by age: ALSWH estimates, compared to other international estimates

			Australian	International			
Prevalence: ALSWH, Australian and International estimates							
Age	ALSWH (identified records)	ALSWH (capture re-capture estimates)	Anstey 2010 (DYNOPTA)	Matthews 2013 (CFAS II, UK)	Lobo 2000 (Europe, Pooled analysis)	Hofman 1991 (Europe, Pooled analysis)	
Follow-up	1996–2012	1996–2012	1991–1994	2008–2011			
80–84	12.8%	16.6%	16.0%	9.5%	12.6%	13.5%	
85–89	22.1% (ages 85–91)	31.0% (ages 85–91)	21.0%	18.1%	20.2%	22.8%	
Incidence: ALSWH and International estimates							
	ALSWH (identified records) per 1000 pyrs	ALSWH (capture re-capture estimates) per 1000 pyrs		Matthews 2016 (CFAS II, UK) per 1000 pyrs	Schrijvers 2012 (Rotterdam Study, 2000) per 1000 pyrs	Fratiglioni 2000 (Europe, Pooled analysis) per 1000 pyrs	Sauvaget 1999 (Northern California) per 1000 pyrs
Follow-up	1996–2012	1996–2012		2008–2011	2000		1980–1988
80–84	24.0	31.9		39.6	24.2 (ages 80–89)	24.1	26.8
85–89	47.8 (ages 85–91)	74.4 (ages 85–91)		55.3 (ages 85+)		53.8	56.8 (ages 85+)

## Discussion

By March 2012, 16% of ALSWH participants who were aged between 71 and 75 in 1996, were recorded as having dementia from the largest single data-source (aged care assessments), and 20% of women were identified from one of the four primary data sources. Using capture–recapture methods the estimated percentage of women who had dementia increased from 20 to 26%. These results highlight the importance of using multiple sources of data, estimating the number people with dementia who may have been missed, and including this ‘undercount’ in the presentation of results. This difference in the estimated prevalence of dementia would have significant implications for the planning and provision of health service needs in older women.

Whilst the methods of identifying records of dementia vary between data sources, the dementia records from the aged care assessment data, cause of death data, and admitted patients hospital data, were all based on doctors’ diagnoses. Dementias recorded with the help of proxies were included in the self-reported dataset, which allowed us to include women who may not have been able to complete the survey alone. However, less than 4% of identified dementia cases were based on self-reported ALSWH records alone. The use of five different sources of dementia notifications strengthens confidence in the analysis and the estimates obtained. The model averaging technique is another strength of the analysis. Using this technique, the results do not dependent on only one model, but are drawn from a number of the best fitting models. This is important, because for the capture–recapture analysis of four and five data sources there were 114 and 6893 possible models, respectively.

Previous research from the ALSWH showed that the probabilistic matching with the National Death Index correctly identified 95% of deaths [21], likewise the age-care data linkage reported high sensitivity and positive predictive values estimates of the sensitivity and PPV above 94% (AIHW communication). This gives confidence in the accuracy of the probabilistic linkage techniques. Aged care assessments identified the largest number of dementia records. Within the age care data, more than one record of dementia was present for 65% of dementia cases identified from this source.

Nevertheless it is possible that the number of dementia records identified from some sources have been overestimated. For example, in hospital records temporary conditions which had similar symptoms could have been misclassified as dementia (e.g., delirium, or other conditions which cause behavioural changes). However, the hospital records were based on doctors’ diagnoses, and 82% of the dementia records identified from the hospital data were also identified from at least one other data source.

Although the ALSWH participants were generally representative of the population of Australian women [11, 16], previous analysis of the 1921–1926 cohort indicated that these women had slightly lower death rates than observed in the general population [30]. If the ALSWH participants were healthier than the general population then the population-wide prevalence of dementia may be underestimated, if ‘healthier’ women were less susceptible to dementia. On the other hand, the prevalence at older ages may be overestimated if the participants’ longer life expectancy increased the age-related risk of dementia.

One of the assumptions of capture–recapture methods is that the population analysed is ‘closed’, with no one entering or leaving. Although in our analysis no additional women entered the study cohort, 44% of the cohort died during the follow-up period. Women leaving the cohort (primarily due to death) may have caused us to underestimate the number of women with dementia. We adjusted for deaths in each age group to reduce the probability of assigning dementia to deceased study participants. This adjustment had only a small effect on the estimates presented.

The use of a defined cohort of women in the analysis meant that the calculation of rates of dementia was straightforward. However, four of the five data sources used were routinely collected administrative records (all except self-reported survey data). As such these sources could potentially be used to estimate rates of dementia at the population level, through data-linkage techniques. This approach would have the advantage of using rates based on the ‘whole population’. However, the assumptions for the capture–recapture methods may be more tenuous if there are difficulties defining the denominator and estimating the number of people entering and leaving the population studied [12].

The rates of incidence and prevalence of dementia for ages below 80 in this analysis were underestimated. Three of the datasets only had records available after the year 2000, so would not have contributed cases identified earlier in the study. For this reason the estimates of dementia prevalence and incidence rates in women aged less than 80 are lower than those reported in other Australian and international studies [3–5, 31–37].

The prevalence and incidence rates of dementia for women aged 80–84 and 85+, based on identified records were broadly consistent with those reported previously, indicating that the estimates gained through linkage of multiple sources are credible (see Table 4) [5, 31–37]. Over the age of 80 estimates based on the capture–recapture techniques were somewhat higher than those estimates published. It is therefore possible that the previously published estimates which did not account for the number of ‘unidentified’ women with dementia are underestimates.

There is evidence from other countries that some types of routinely collected data, such as United States Medicare claims (which do not have universal coverage, and cover a different range of services from the Australian Medicare), may overestimate the prevalence of dementia [38], so the use of some of the multiple linked data sources may have inflated these estimates, compared to other studies which used clinical assessments on all study participants [39, 40]. However, a recent UK study found dementia recorded in hospital admission data, agreed well with primary care records of dementia [41].

Other studies of dementia have used measures such as Mini-Mental State Examination, the Geriatric Mental State—Automated Geriatric Examination for Computer Assisted Taxonomy diagnosis algorithm, or an interview or clinical assessment to define dementia [3, 34, 36, 39, 40]. The current study uses more diverse assessments of dementia collected from 5 separate data sources. However, the rates we present give estimates of older women identified as having dementia in different health care settings.

The use of existing linked data to identify people living with dementia, as demonstrated in this study, has clear advantages in large population based studies over separate study-specific individual clinical assessments to determine diagnoses. For the purposes of public policy and planning of health services these methods can provide population-level estimates as well as sub-population comparisons (e.g., between urban and rural areas and for socially disadvantaged groups) and trends over time.

## Conclusions

This study demonstrates using routinely collected health data with record linkage and capture–recapture methods can produce plausible estimates for dementia prevalence and incidence.

## Additional files

**Additional file 1.** Tables A1 to A5.

**Additional file 2.** Dementia prevalence and incidence by single year of age (dataset).

## Abbreviations

ALSWH: Australian Longitudinal Study on Women’s Health
ATC: Anatomical Therapeutic Chemical index
CI: confidence interval
ICD: International Classification of Diseases
