# Supplementary material for: Estimating the prevalence of dementia using multiple linked administrative health records and capture–recapture methodology
Source: Emerg Themes Epidemiol. 2017 Feb 27;14:3. doi: 10.1186/s12982-017-0057-3 (PMC5327574; doi:10.1186/s12982-017-0057-3)
Supplement: Supplementary file 1 — Additional file 1. Tables A1 to A5. [file 12982_2017_57_MOESM1_ESM.docx]

Table A1: Codes used to identify dementia

| Anatomical Therapeutical Chemical (ATC) code | Drug name |
| --- | --- |
| N06DA01 | Tacrine |
| N06DA02 | Donepezil |
| N06DA03 | Rivastigmine |
| N06DA04 | Galantamine |
| N06DA05 | Ipidacrine |
| N06DA52 | Donepezil and Memantine |
| N06DA53 | Donepezil, Memantine and Ginkgo folium |
| N06DX01 | Memantine |
|  |  |
| Aged Care Assessment Program Health condition codes | Health condition |
| & Aged Care Funding Instrument Medical diagnoses |  |
| **0500** | Dementia in Alzheimer’s disease |
| 0501 | Dementia in Alzheimer’s disease with early onset (<65 yrs) |
| 0502 | Dementia in Alzheimer’s disease with late onset (>65 yrs) |
| 0503 | Dementia in Alzheimer’s disease, atypical or mixed type |
| 0504 | Dementia in Alzheimer’s disease, unspecified |
| **0510** | Vascular dementia |
| 0511 | Vascular dementia of acute onset |
| 0512 | Multi-infarct dementia |
| 0513 | Subcortical vascular dementia |
| 0514 | Mixed cortical & subcortical vascular dementia |
| 0515 | Other vascular dementia |
| 0516 | Vascular dementia – unspecified |
| **0520** | **Dementia in other diseases classified elsewhere** |
| 0521 | Dementia in Pick’s disease |
| 0522 | Dementia in Creutzfeldt-Jakob disease |
| 0523 | Dementia in Huntington’s disease |
| 0524 | Dementia in Parkinson’s disease |
| 0525 | Dementia in human immunodeficiency virus (HIV) disease |
| 0526 | Dementia in other specified diseases classified elsewhere |
| **0530** | **Other dementia** |
| 0531 | Alcoholic dementia |
| 0532 | Unspecified dementia (includes presenile & senile dementia) |
|  |  |
| ICD9 codes |  |
| 2900 | Senile dementia, uncomplicated |
| 29010 | Presenile dementia, uncomplicated |
| 29040 | Arteriosclerotic dementia, uncomplicated |
| 2912 | Other alcoholic dementia |
| 2941 | Dementia in conditions classified elsewhere |
| 2949 | Unspecified organic brain syndrome (chronic) |
| 3310 | Alzheimer's disease |
|  |  |
| ICD10 codes |  |
| F00 | Dementia in Alzheimer's disease |
| F01 | Vascular dementia |
| F02 | Dementia in other diseases classified elsewhere |
| F03 | Unspecified dementia |
| F107 | Mental and behavioural disorders due to use of alcohol- Residual and late-onset psychotic disorder |
| G30 | Alzheimer's disease |

NB. Ginkgo folium (N06DX02), which can take the form of a dietary supplement, was not used as an identifier of dementia cases

Table A2: Formulae used for model averaging and to adjust for deaths in each age group

| Equation number | Formulae | Notes |
| --- | --- | --- |
| 1 |  |  |
|  | $Quasi Akaike^{'}s Information Criterion=QAICc=-\left[ \frac{2L}{c} \right]+2K+\frac{2K(K+1)}{n-K-1}$ | [29] pages 69-70 |
|  |  |  |
|  | where $L=Log likelihood$ |  |
|  |  |  |
| 2 | $c=overdispersion parameter=\frac{deviance}{degrees of freedom}$ | Based on model with lowest QAICc. If $c<1$, then set $c=1$ |
|  |  |  |
|  | and $K=number of parameters in the model+1, to account for c$ |  |
|  |  |  |
| 3 | $Weight for each model=w_{i}=\frac{e^{-\Delta QAICc/2}}{\sum e^{-\Delta QAICc/2}}$ | [28]  where $\Delta QAICc$ is the difference in $QAICc$ between a particular model and the model with the lowest $QAICc$ and the sum is over all models |
|  |  |  |
| 4 | $Weighted Estimate=\hat{\bar{\theta}}=\sum_{i=1}^{R} w_{i}\hat{\theta_{i}}$ | Estimate of number of unidentified dementia cases, where $\hat{\theta_{i}}$ is the estimate from each of the candidate models |
|  |  |  |
| 5 | $Standard error of estimate=se\left( \hat{\theta} \right)=\sum_{i=1}^{R} w_{i}\sqrt{var(\hat{\theta}_{i})+{(\hat{\theta}_{i}-\bar{\theta})}^{2}}$ | [29] pages 162-163. $var(\hat{\theta}_{i})$ estimated from each of the candidate models |
|  |  |  |
| 6 | $S_{j}=Scalar adjustment for deaths in each age group$  $=1-\frac{Number of cases who died before the median age of death in that age group}{Number of cases in age group}$ | $\hat{\theta_{j}}\times S_{j}=Estimate adjusted for deaths$ |
| 7 | $Pooled estimate across age groups= \sum_{j=1}^{A} \left[ \hat{\theta_{j}}\times S_{j} \right]$ |  |
| 8 | $Standard error of estimate pooled across age groups=\sqrt{\sum_{j=1}^{A} \left[ \left\langle exp( {\hat{\bar{\theta}}}_{j}) \right\rangle^{2}{{\times se(\theta}_{j})}^{2}\times S_{j}^{2} \right]}$ | Based on Taylor series expansion |

Table A3: *Number of new records of dementia by age identified by different combinations of four data sources for the States of Queensland, New South Wales and South Australia (n=7750)*

|  |  |  | Age |  |  |  |  |
| --- | --- | --- | --- | --- | --- | --- | --- |
|  | 68-78 | 79-80 | 81-82 | 83-84 | 85-86 | 87-91 | Total |
| A | 20 | 14 | 7 | 7 | 4 | 12 | 64 |
| B | 6 | 25 | 58 | 144 | 195 | 182 | 610 |
| C | 18 | 18 | 26 | 26 | 23 | 3 | 114 |
| D | 6 | 4 | 15 | 14 | 16 | 18 | 73 |
| AB | 7 | 10 | 14 | 20 | 21 | 11 | 83 |
| AC | 14 | 6 | 0 | 3 | 1 | 0 | 24 |
| AD | 2 | 2 | 1 | 2 | 3 | 3 | 13 |
| BC | 3 | 14 | 26 | 36 | 23 | 12 | 114 |
| BD | 19 | 44 | 50 | 69 | 53 | 30 | 265 |
| CD | 9 | 7 | 4 | 2 | 0 | 0 | 22 |
| ABC | 3 | 1 | 3 | 2 | 1 | 0 | 10 |
| ABD | 7 | 22 | 14 | 13 | 12 | 4 | 72 |
| ACD | 2 | 0 | 2 | 0 | 0 | 0 | 4 |
| BCD | 14 | 19 | 22 | 14 | 5 | 0 | 74 |
| ABCD | 7 | 6 | 6 | 1 | 1 | 0 | 21 |
|  |  |  |  |  |  |  |  |
| Identified number | 137 | 192 | 248 | 353 | 358 | 275 | 1563 |
| Estimated extra | 19 | 27 | 56 | 79 | 111 | 100 | 438 |
| Total=identified + extra | 156 | 219 | 304 | 432 | 469 | 375 | 2001 |
| 95% CI | 139, 173 | 195, 243 | 263, 346 | 369, 486 | 232, 701 | 294, 456 | 1768, 2499 |
|  |  |  |  |  |  |  |  |
| Adjustment for deaths | 1-(26/137)=0.81 | 1-(13/192)=0.93 | 1-(23/248)=0.91 | 1-(29/353)=0.92 | 1-(27/358)=0.92 | 1-(30/275)=0.89 |  |
|  |  |  |  |  |  |  | Pooled estimate  95% CI |
|  |  |  |  |  |  |  | 1955  1696, 2214 |

*Correction factor of 0.125 added to cells which contain no identified cases*

A = Self-reported survey data

B = Aged care data

C = Death certificate data

D = Prescription data

Table A4: *Number of new records of dementia by age as identified by different combinations of five data sources for the States of Queensland, New South Wales and South Australia (n=7750)*

|  |  |  | Age |  |  |  |  |
| --- | --- | --- | --- | --- | --- | --- | --- |
|  | 68-78 | 79-80 | 81-82 | 83-84 | 85-86 | 87-91 | Total * |
| A | 14 | 8 | 6 | 3 | 3 | 10 | 44 |
| B | 4 | 7 | 23 | 70 | 98 | 105 | 307 |
| C | 12 | 14 | 10 | 15 | 12 | 12 | 75 |
| D | 2 | 3 | 11 | 9 | 12 | 14 | 51 |
| E | 28 | 19 | 27 | 32 | 34 | 35 | 175 |
| AB | 2 | 0 | 9 | 8 | 13 | 7 | 39 |
| AC | 7 | 2 | 0 | 2 | 0 | 1 | 12 |
| AD | 2 | 1 | 1 | 2 | 2 | 3 | 11 |
| AE | 9 | 4 | 0 | 6 | 0 | 0 | 19 |
| BC | 0 | 6 | 11 | 20 | 17 | 10 | 64 |
| BD | 6 | 16 | 21 | 21 | 23 | 16 | 103 |
| BE | 13 | 16 | 38 | 63 | 72 | 61 | 263 |
| CD | 4 | 2 | 2 | 1 | 0 | 0 | 9 |
| CE | 18 | 14 | 8 | 8 | 5 | 2 | 55 |
| DE | 4 | 3 | 2 | 7 | 4 | 5 | 25 |
| ABC | 1 | 1 | 2 | 1 | 0 | 1 | 6 |
| ABD | 1 | 4 | 5 | 4 | 6 | 2 | 22 |
| ABE | 5 | 9 | 7 | 9 | 7 | 3 | 40 |
| ACD | 1 | 0 | 1 | 0 | 0 | 0 | 2 |
| ACE | 7 | 4 | 0 | 1 | 1 | 0 | 13 |
| ADE | 0 | 1 | 0 | 0 | 1 | 0 | 2 |
| BCD | 5 | 5 | 10 | 6 | 1 | 0 | 27 |
| BCE | 13 | 14 | 16 | 25 | 17 | 10 | 95 |
| BDE | 14 | 23 | 26 | 41 | 25 | 13 | 142 |
| CDE | 6 | 4 | 2 | 1 | 0 | 0 | 13 |
| ABCD | 2 | 3 | 2 | 0 | 0 | 0 | 7 |
| ABCE | 2 | 2 | 2 | 1 | 1 | 0 | 8 |
| ABDE | 6 | 16 | 6 | 8 | 5 | 2 | 43 |
| ACDE | 1 | 0 | 1 | 0 | 0 | 0 | 2 |
| BCDE | 12 | 15 | 17 | 14 | 9 | 0 | 67 |
| ABCDE | 5 | 7 | 5 | 2 | 2 | 0 | 21 |
|  |  |  |  |  |  |  |  |
| Identified number | 206 | 223 | 271 | 380 | 370 | 312 | 1762 |
| Estimated extra | 21 | 20 | 65 | 43 | 84 | 92 | 374 |
| Total ^A^  95% CI | 227  215, 240 | 243  233, 253 | 336  282, 390 | 423  402, 444 | 454  401, 508 | 404  356, 452 | 2136  2031, 2281 |
|  |  |  |  |  |  |  |  |
| Adjustment for deaths | 1-(22/206)  =0.89 | 1-(17/223)  =0.92 | 1-(16/271)  =0.94 | 1-(29/380)  =0.92 | 1-(24/370)  =0.94 | 1-(40/312)  =0.87 |  |
|  |  |  |  |  |  |  | Pooled estimate  95% CI |
|  |  |  |  |  |  |  | 2088  1995, 2182 |

*Correction factor =0.0625 added to cells which contain no identified cases*

* Total=observed + extraA = Self-reported survey data

B = Aged care data

C = Death certificate data

D = Prescription data

E = Hospital admission patients data (only for States of Queensland, New South Wales, South Australia; Total n=7750)
